# A nationwide student survey on biochemistry learning resources in Italy

**DOI:** 10.1002/2211-5463.70355

**Published:** 2026-10-16

**Authors:** Anna Marabotti, Ivano Eberini, Maria Luisa Genova, Francesca Mensitieri, Francesco Malatesta

**Affiliations:** ^1^ SIB Education Group Committee Italy; ^2^ Dipartimento di Chimica e Biologia “A. Zambelli” Università degli Studi di Salerno Fisciano Italy; ^3^ Dipartimento di Scienze Farmacologiche e Biomolecolari “Rodolfo Paoletti” Università degli Studi di Milano Italy; ^4^ Dipartimento di Scienze Biomediche e Neuromotorie Alma Mater Studiorum‐Università di Bologna Italy; ^5^ Dipartimento di Medicina, Chirurgia e Odontoiatria “Scuola Medica Salernitana” Università degli Studi di Salerno Baronissi Italy; ^6^ Dipartimento di Scienze Biochimiche Sapienza Università di Roma Italy

**Keywords:** biochemistry education, learning materials, student learning behaviors, student perceptions, teaching and learning resources

## Abstract

Biochemistry is widely recognized as a crucial discipline in life sciences. However, learning biochemistry poses significant challenges, requiring students to understand complex molecular concepts while integrating knowledge across multiple disciplines. This necessitates instructors to innovate their teaching of biochemistry, considering students' learning behaviors. Here, we present the results of a nationwide survey of university students, conducted by the Education Group of the Italian Society of Biochemistry and Molecular Biology, from more than 2000 students across 49 academic institutions in Italy, with the aim of capturing not only which learning resources are relied on but also the underlying motivations for their choices and perceptions of biochemistry. Results show that overall, biochemistry is perceived as an interesting subject to study, even if challenging. Students primarily rely on instructor‐provided materials, such as lecture slides, as well as self‐acquired resources, including audio recordings and personal notes. Textbooks are used mainly as references when the preferred materials above do not sufficiently support students' learning. AI tools are increasingly used but not yet preferred. This strong reliance on teacher guidance underscores the need for high‐quality teaching materials and greater integration of practical, application‐oriented learning activities. By mapping these trends, our survey provides a foundation for critical reflection on current teaching practices and offers insights that may guide the development of more effective learning tools. Our broader goal is to support educators in creating environments that foster deeper understanding, sustained engagement, and a more inclusive learning experience in biochemistry.

## Graphical abstract

We conducted a survey among Italian university students to investigate their preferred study materials for biochemistry. We then analyzed the collected data to identify these materials. This information may be valuable to the Biochemistry educational community. The graphical abstract was created with Claude (Opus 5.5; Anthropic, 2026).

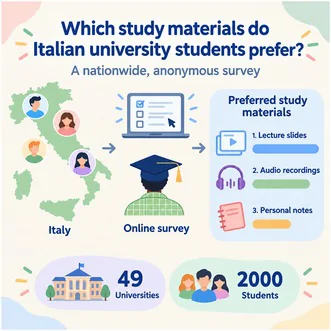

**Abbreviations**
GD‐SIB: Gruppo Didattica SIB (Education Group of the Italian Society of Biochemistry and Molecular Biology)
STEM: Science, Technology, Engineering and Mathematics

Biochemistry is a fundamental discipline within medicine, life sciences, pharmacy, chemistry, and related fields. It provides the conceptual framework for interpreting biological phenomena at the molecular level, bridging chemistry and biology to explain the mechanisms that sustain life [1]. Because of this central role, biochemistry is an essential component of undergraduate and graduate curricula in many scientific, medical, and allied health degree programs.

Biochemistry education has traditionally relied on printed textbooks, many of which—having undergone multiple international editions by major academic publishers—have become widely used reference sources for foundational concepts among students and teachers worldwide. Notably, textbooks such as *Lehninger Principles of Biochemistry* [2], *Biochemistry* [3], *Harper's Illustrated Biochemistry* [4], and *Fundamentals of Biochemistry* [5] exemplify enduring, widely adopted resources that standardize the representation of complex molecular processes and structure biochemistry curricula in medicine and the broader STEM fields. While these textbooks provided rigor and consistency, opportunities for interactive and meaningful engagement with the material were largely confined to end‐of‐chapter questions, as was the case in earlier editions published up to a few years ago. Learning followed a predominantly linear, instructor‐centered model, with students relying on fixed diagrams and dense prose, supplementing their handwritten notes with whatever materials lecturers chose to distribute, most often lecture slides.

More recently, the need for real‐time visualization and rapid updates in an increasingly fast‐evolving discipline has highlighted the cognitive burden placed on learners, who must translate two‐dimensional illustrations into dynamic three‐dimensional biochemical systems. To mitigate these challenges, traditional instruction has been complemented by tangible aids, such as physical and digital molecular models, to enhance spatial visualization and conceptual understanding [6]. Over time, the integration of online repositories, interactive simulations, and digital platforms such as Proteopedia [7] has further reshaped the learning environment, signaling a shift toward pedagogical frameworks that prioritize active engagement, interactivity, and increased learner autonomy. This evolution reflects a broader shift from transmissive teaching toward constructivist pedagogical approaches that emphasize active knowledge construction and posit that learners build new knowledge upon their existing cognitive frameworks, not by passively absorbing information but by actively making sense of it [8, 9, 10].

Despite its importance and innovations in teaching technologies and methodologies, biochemistry remains a demanding subject. The discipline requires the integration of abstract molecular concepts with experimental evidence and clinical or biological applications, posing challenges for both learners and instructors [11]. In this context, the quality and accessibility of study materials and their alignment with intended learning outcomes can strongly influence students' learning strategies and subsequent performance in summative assessments. Understanding how students approach the study of biochemistry and which resources they consider most useful is therefore highly relevant for educators. Although individual instructors may have insights into their students' preferences, systematic studies across institutions remain limited.

Two studies examining the educational resources used by medical students were conducted in 2012 and in 2019 [12, 13]. In the first article, 60 first‐year medical students at an Indian university (Christian Medical College, Vellore, India) were surveyed to assess the impact of the e‐resources on various aspects of their learning in biochemistry. The article reports that the materials available to those students for studying biochemistry include textbooks, lecture slides, lecture notes, study materials for practicals, and a case‐based tool for self‐assessment, but focuses exclusively on the use of e‐learning resources. The results showed that students believed e‐learning resources had a positive impact on various aspects of their learning of biochemistry, and the vast majority recommended that they be retained as part of the course. Of course, this study has limitations due to the extremely small sample size and its specific focus (students from faculties other than medicine were not included). In the second article, a sample of 350 undergraduate and postgraduate medical students from two major Australian universities (Sydney and New South Wales) answered a survey designed to assess the frequency with which they draw on various resources, both when approaching new material and when reviewing content already covered. From this study, it emerged that taking notes and reading textbooks were the most commonly used resources. Additionally, most students confirmed the use of a variety of e‐learning tools. In this case, the sample analyzed was larger, but still confined to medical schools, and the questionnaire surveyed students on the frequency of their resource utilization for learning new concepts or reviewing past material, not specifically on learning biochemistry.

More recently, a survey conducted on a sample of 135 first‐ and second‐year undergraduate students at universities in Spain and the United Kingdom examined their strategies and habits for studying science subjects, to evaluate the extent of their metacognitive awareness [14]. From their survey, Fergus and coworkers indirectly inferred that students use notes and textbooks, as well as other materials such as lecture slides and flashcards, but their preferences for one material or the other are not addressed.

Perhaps, the most comprehensive review on methodologies and teaching resources about Biochemistry learning was conducted by Silva and coworkers [11] by analyzing a total of 271 articles from the ERIC and SciFinder databases, published between 2012 and 2024. 45.4% of studies were conducted in the United States, while a lower percentage came from China (9.6%), India (6.6%), Brazil (4.4%), and Canada (4.4%). No data were explicitly reported about the percentage of articles from EU countries. The teaching resources considered in this study, however, were mostly innovative tools such as digital tools, models and games, online activities, multimedia approaches, and immersive technology, whereas more traditional materials were not analyzed in depth.

Within the Italian context, an article published in 2019 by colleagues who, like us, are members of the Italian Society of Biochemistry and Molecular Biology (SIB) discussed the challenges of presenting traditional biochemical topics to a student body with highly varied backgrounds and academic interests [15]. The data were collected in the frame of Biochemistry courses compulsory for MS curricula in Food Science and Technology and in Catering Science and Technology at the University of Milan, over the three academic years from 2016 to 2018. However, the data were not collected through a purpose‐designed survey but were instead drawn from standard course satisfaction questionnaires completed by students. Those questionnaires did not include specific questions regarding course materials; therefore, to the best of our knowledge, this specific aspect of the materials used to study biochemistry has not previously been addressed in the literature available to us.

Building on this context, in 2025, as members of the Coordinating Committee of the Italian Society of Biochemistry and Molecular Biology Education Group (Gruppo Didattica SIB, hereafter GD‐SIB), we conducted a national survey to explore the study practices of university students in Italy. The results of our study provide a broad perspective on the educational resources currently used in biochemistry courses in Italy. We believe it is important to bring these findings to the attention of the international scientific community, to supplement the existing literature and to encourage other national and international organizations to carry out similar analyses, with the aim of stimulating reflection on how teaching and learning in a core discipline such as biochemistry may be improved.

## Methods

With the aim of testing and refining a methodological approach, a pilot questionnaire was preliminarily administered at the end of 2024 to students at the University of Salerno enrolled in two different degree courses: Chemistry and Environmental Sciences. That original pilot questionnaire was anonymous and asked students to indicate their three favorite study materials from a list of options and to explain the reasons for each choice. Despite the small sample size (approx. 50 students), common choices and their underlying reasons were identified and discussed with the students, demonstrating the usefulness of the pilot study (unpublished data).

Based on such preliminary results, we decided to expand the questionnaire, administering it to a larger sample of students and including a broader analysis of other aspects as well, for example to investigate whether there were correlations between the use of specific study materials and the students' examination results, or even to ascertain whether students enrolled in different degree programs exhibit different study habits in their use of learning materials. As a result of careful collaborative design work, a new questionnaire was developed that was considerably more comprehensive than the pilot version. Like the pilot one, our new questionnaire is completely anonymous and includes no items pertaining to any personal, sensitive, medical, or clinical data of the participants. This study has been approved by the Ethics Committee of the University of Milan (document 67/2026). For the purposes of this study, General Biochemistry is defined as a course covering the structure and function of biological macromolecules and the major metabolic pathways.

The questionnaire is organized into several sections (Table 1). Section 1 collects geographic and academic information from all participants. Section 2 collects specific information on examination marks and was presented only to respondents who reported they had already passed the General Biochemistry examination (Question 5, option A). The following sections (i.e., Sections 3–7) were presented separately to students who reported having passed the General Biochemistry course in their current degree program or in a previous degree program, and to students who reported that they were actively preparing to take the examination (i.e., respondents who selected options A, B, or E in Question 5). Students who had taken the course but were not yet preparing for the examination, along with those who had not taken the course, were excluded, as they were unlikely to provide useful information for our study.

**Table 1:** Description of the custom‐designed questionnaire administered anonymously to students, available in Italian and English.

Question	Format	Possible answers	Mandatory
**Section 1 (for all participants)**			
(1) select the university in which you are enrolled	Drop‐down menu containing 66 names of Italian universities + ‘other university’	One of the universities listed in the menu	Yes
(2) If you answered “Other University” indicate here the name of your university	Free text	Free answer	No
(3) What kind of degree program are you pursuing?	Multiple‐choice answer	One among: 3‐years degree Master's degree Single cycle degree	Yes
(4) Which scientific area is your degree course in?	Multiple‐choice answer	One among: Agriculture Biology and Biotechnology Chemistry Pharmacy Mathematics and Physics Medicine and Allied Health Natural and Environmental Sciences Veterinary Other (specify)	Yes
(5) Does your current degree program include an examination in General Biochemistry?	Multiple‐choice answer	One among^a^: Yes, I have taken the course and already passed the examination Yes, I have taken the course and have prepared/are preparing for the examination but have not yet taken/passed it Yes, I have taken the course, but I am not preparing for the examination Yes, but I haven't taken the course yet. No, but I passed it in my previous degree program No	Yes
**Section 2 (only for respondents who selected option “a” in question 5)**			
(6) What grade did you pass the examination with?	Multiple‐choice answer	One among: 18–21/30 22–24/30 25–27/30 28–30/30 cum laude	Yes
**Sections 3–4 (for respondents who answered options a, b, or e in question 5)** ^b^			
(7) Indicate the material or instrument that you think was the most important in preparing for the General Biochemistry examination	Multiple‐choice answer	One among: Personal notes Notes from classmates Recording of notes eventually processed later Slides provided by the lecturer Textbook(s) recommended by the lecturer in hard copy Textbook(s) recommended by the lecturer in e‐book format Other general biochemistry texts not recommended by the lecturer Video lectures recorded by the lecturer Internet videos or tutorials E‐learning courses Miscellaneous materials (notes, slides) downloaded from Internet sites ChatGPT or other AI tools Other (specify)	Yes
(8) Why do you think this material you indicated was the most important for your preparation?	Free text	Free answer	Yes
(9) Indicate the material or instrument that you think was the second most important in preparing for the General Biochemistry examination	Multiple‐choice answer	One among: Personal notes Notes from classmates Recording of notes eventually processed later Slides provided by the lecturer Textbook(s) recommended by the lecturer in hard copy Textbook(s) recommended by the lecturer in e‐book format Other general biochemistry texts not recommended by the lecturer Video lectures recorded by the lecturer Internet videos or tutorials E‐learning courses Miscellaneous materials (notes, slides) downloaded from Internet sites ChatGPT or other AI tools I did not use any other material Other (specify)	Yes
(10) Why do you think this material you indicated was the second most important for your preparation?	Free text	Free answer	Yes
(11) Indicate the material or instrument that you think was the third most important in preparing for the General Biochemistry examination	Multiple‐choice answer	One among: Personal notes Notes from classmates Recording of notes eventually processed later Slides provided by the lecturer Textbook(s) recommended by the lecturer in hard copy Textbook(s) recommended by the lecturer in e‐book format Other general biochemistry texts not recommended by the lecturer Video lectures recorded by the lecturer Internet videos or tutorials E‐learning courses Miscellaneous materials (notes, slides) downloaded from Internet sites ChatGPT or other AI tools I did not use any other material Other (specify)	Yes
(12) Why do you think this material you indicated was the third most important for your preparation?	Free text	Free answer	Yes
(13) What other kind of material would you have liked to have had available to study General Biochemistry?	Free text	Free answer	No
**Sections 5–6 (for respondents who answered options a, b, or e in question 5)** ^c^			
(14) On average, approximately how many hours a day did you study/are you studying to prepare for the General Biochemistry examination?	Multiple choice	One among: 1–2 3–4 5–6 d 7–8 more than 8	Yes
(15) And approximately how many weeks (studying on average 5 days a week for the hours you stated in the previous question) did it take you to feel sufficiently prepared/do you think you need to feel sufficiently prepared to take the General Biochemistry examination?	Multiple choice	One among: 1–2 3–4 5–6 d 7–8 more than 8	Yes
(16) How important do you think it was/it is to take the General Biochemistry course to pass the examination?	Likert scale	1 to 5 (1: not at all; 5: very much)	Yes
(17) Beyond the grade you got/you will get, how much did you enjoy studying General Biochemistry?	Likert scale	1 to 5 (1: not at all; 5: very much)	Yes
(18) What would have increased/would increase your liking for General Biochemistry? (If you indicated a score of 5 in the previous question, you may not answer this one)	Free text	Free answer	No
**Section 7 (for respondents who answered options a, b, or e in question 5)**			
(19) What are the attendance directions for the General Biochemistry course you have taken/are taking?	Multiple‐choice answer	One among: Mandatory attendance (with attendance tracking methods) Recommended attendance No indication	Yes
(20) Does the General Biochemistry course you have taken/are taking involve only lectures or also practical laboratory exercises or activities?	Multiple‐choice answer	One among: Face‐to‐face lectures only Also practical laboratory exercises or activities	Yes
(21) In your opinion, how important is it to know General Biochemistry for your course of study?	Likert scale	1 to 5 (1: not at all; 5: very much)	Yes
(22) If you had to identify one difficulty in the study of General Biochemistry, which of these three do you think is the greatest?	Multiple‐choice answer	One among: The concepts are not immediately understandable There are many concepts to understand, but they are quite understandable There are not many concepts to understand, but it is necessary to study everything by heart	Yes

^a^ All those who answered options a, b, and e for question 5 continue with the following questions; the others finish the questionnaire

^b^ Sections 3 and 4 contain the same questions but have been kept separate in order to collect responses separately from people who have already passed the examination and those who have not yet passed it

^c^ Sections 5 and 6 contain the same questions but have been kept separate in order to collect responses separately from people who have already passed the examination and those who have not yet passed it.

Sections 3 and 4 focus on study materials. Respondents were asked to indicate, in order of importance, the three materials they considered most relevant for their preparation, and to justify each choice in free‐text responses. These sections also collect additional information on study experience (including a quantitative estimate of total workload in terms of daily intensity and overall duration), course attendance (either mandatory or recommended), practical activities, and students' perceived relevance and difficulty of the course, distinguishing between conceptual complexity (e.g., material not immediately understandable), breadth of content (e.g., many concepts to learn, but generally straightforward to understand), and memorization demands (e.g., the reliance on rote memorization for vast amounts of information). Most of these questions were designed and included in the survey following discussions within the GD‐SIB Coordinating Committee and informal feedback from students during the presentation of the pilot questionnaire results mentioned above. As this is an exploratory, descriptive survey aimed at capturing observable study practices (what students actually do), no formal psychometric validation was conducted prior to administration.

The present questionnaire was initially developed in Italian and launched on 25 March 2025, using the Google Forms platform. Following requests from some teachers, an English version was released on 4 April 2025, on the same platform to accommodate foreign students enrolled in Italian universities. Both versions remained open until 30 June 2025. The anonymous bilingual survey was distributed by SIB members holding Biochemistry chairs in life science courses across Italy, who shared it with their students via a QR code link.

Once the questionnaires had closed, the responses were automatically saved by the platform in a .csv file and analyzed using Microsoft Excel functions. Free‐text responses, provided by students to justify their choice of study materials, were analyzed qualitatively using an inductive approach. Responses were read iteratively, grouped into recurring meaning units, and then clustered into broader thematic categories. These themes were refined through discussion and compared interpretations among the authors until consensus was reached to reduce individual bias. Given the exploratory nature of the study, this approach was deemed appropriate for capturing the diversity of student perspectives.

Beyond the descriptive analysis of the results, statistical testing was conducted to identify significant differences in the use of materials among the student population, with a specific focus on two groups. Group A (Table 2) was designed to examine whether students with different levels of academic performance and/or examination preparation used different study materials. The following subgroups were considered: students who passed the biochemistry examination with marks ≥ 28/30 (The Italian university grading system is based on a 30‐point scale for individual examinations. The minimum grade to pass an examination is 18/30. The highest standard grade is 30/30. ‘30 *cum laude*’, that is, ‘with honors’ is awarded for outstanding performances) (Table 2, group A1); students who passed the examination with marks < 28/30 (Table 2, group A2); and students who had not yet received a mark, either because they were still preparing for the examination or because they had failed it (One of the distinctive features of the Italian university system is the flexibility in examination retakes: if students fail an examination, they can generally retake it during the next available session. There is no formal limit on the number of attempts before graduation) (Table 2, group A3). For this first case, the *χ* ^2^ test of independence was applied to contingency tables in which rows represent the study materials considered for the statistical analysis, and columns represent the three choice positions (i.e., 1st, 2nd, and 3rd ranked choice positions). To assess the strength of association among categorical variables, Cramér's *V* was calculated [16].

**Table 2:** Statistical analyses performed on different groups of students. (1) The corrected χ^2^ was obtained by dividing the raw χ^2^ statistic by an inflation factor of 3, which reflects the fact that each student contributed one count to each of the three choices, making the apparent sample size three times the true number of independent units; the corrected *P*‐value was then derived from this corrected χ^2^ under the χ^2^ distribution with the same degrees of freedom. (2) Cramér's *V* was computed as $\sqrt{\chi^{2}/\left(n\left(k-1\right)\right)}$, where n is the true number of independent units (students) and k = 3 is the minimum dimension of the 10 × 3 contingency table. (3) The Bonferroni‐corrected *P*‐value was obtained by multiplying the raw *P*‐value from each pairwise χ^2^ test by 3, which is the number of pairwise comparisons made. (4) Cramér's *V* was computed as in (2). In this case, n is the combined sample size of the two disciplines and k = 2 is the minimum dimension of the contingency table.

(A) Is the preference for the material correlated with preparation/performance of the students?					
Code	Group	*n*	Corrected χ^2^ (1)	Corrected *P*‐value	Cramér's *V* (2)
A1	Passed with mark ≥ 28/30	434	139.06	9.72 × 10^−21^	0.400
A2	Passed with mark < 28/30	454	89.26	1.96 × 10^−11^	0.314
A3	Preparing/not yet passed	795	123.08	1.10 × 10^−17^	0.278

(B) Is the preference for the material correlated with different disciplines?					
Code	Group	*n*	χ^2^	Corrected *P*‐value (3)	Cramér's *V* (4)
B1 1^st^ choice	Biology vs Medicine	1155	79.79	5.34 × 10^−13^	0.263
	Biology vs Pharmacy	784	21.65	3.02 × 10^−2^	0.166
	Medicine vs Pharmacy	849	17.89	1.09 × 10^−1^	0.145
B2 2^nd^ choice	Biology vs Medicine	1155	40.34	1.98 × 10^−5^	0.187
	Biology vs Pharmacy	784	9.29	1.00	0.109
	Medicine vs Pharmacy	849	44.48	3.45 × 10^−6^	0.229
B3 3^rd^ choice	Biology vs Medicine	1155	31.43	7.51 × 10^−4^	0.165
	Biology vs Pharmacy	784	14.35	3.31 × 10^−1^	0.135
	Medicine vs Pharmacy	849	32.46	4.96 × 10^−4^	0.196

Group B (Table 2) examines pairwise comparisons between students enrolled in the following degree programs: Biology vs Medicine and Allied Health, Biology vs Pharmacy, and Medicine and Allied Health vs Pharmacy, respectively. For this second case, a *χ* ^2^ test of independence was performed for each pairwise comparison. The contingency tables mapped study materials in the rows and the two disciplines in the columns. The Bonferroni correction was applied in this case, and Cramér's *V* was computed for each pairwise comparison. All *χ* ^2^ values and *P*‐values were independently verified by MathWorks Matlab.

## Results

We received 2012 valid questionnaire submissions, of which 1885 were from the Italian version and 127 were from the English version, spanning 49 universities. Most responses came from three‐year and single‐cycle degree programs in nearly equal proportions (44% and 42%, respectively), while only 14% came from master's degree students. This outcome was expected, since coursework in General Biochemistry in Italy is predominantly offered within three‐year and single‐cycle degree programs, with master's degree programs emphasizing more specialized content.

Figure 1 illustrates the geographic distribution of responses across Italy. In general, response rates aligned with institution size, with larger universities providing more responses, particularly for the Italian‐language questionnaire. However, we cannot rule out the possibility that other factors, such as the higher number of SIB members at these universities and more effective communication of the importance of the questionnaire to students, may have contributed to this imbalance in the distribution of responses. In particular, the State Universities of Milan, Salerno, Bologna, Rome ‘Tor Vergata’, and Catania had the highest response rates, each exceeding 100 responses and collectively accounting for 36% of the total. In terms of response provenance, 45.5% of responses from bachelor students and 44.6% from master students were concentrated across those five universities, whereas those same five institutions contributed a mere 19.8% of the single‐cycle degree data.

**Fig. 1:**
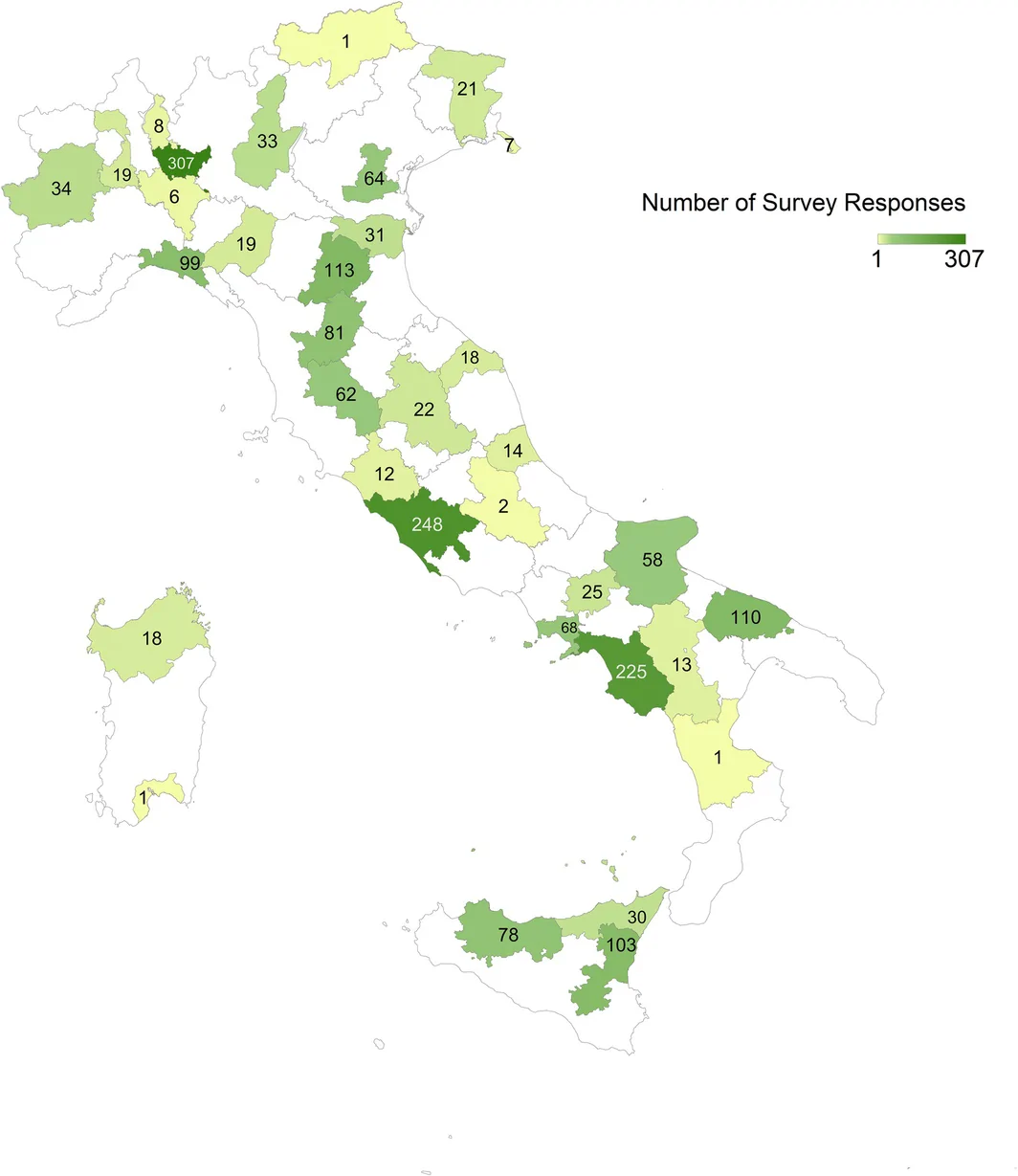
Number of survey responses across Italy. Colored areas represent the provinces– administrative areas within regions– where the 49 participating universities are located (each province can host more than one university).

Around 6% of the total responses were received from international students studying in Italy through the English‐language questionnaire. We noticed that almost 70% of such students were enrolled in Medicine and Allied Health degree programs. It should also be noted that the distribution of degree programs taught in English across Italian Universities is uneven, with such programs considerably less widespread throughout the country. A comparative analysis of responses regarding 1st‐choice study materials in the English versus Italian versions of the survey showed a significantly different distribution between the two groups (χ^2^ = 93.94, *P* < 0.0001). The effect size is moderate (Cramér's *V* = 0.26), reflecting a genuine and meaningful divergence in study habits. However, due to the limitations of this specific subsample, the responses from foreign students studying in Italy were not analyzed separately in greater depth but were instead aggregated with those of the Italian cohort. We intend to investigate this aspect further in future studies, possibly by expanding the international student sample.

Figure 2 shows the distribution of the responders across different scientific areas, as defined by their degree programs: around 34% of responses came from students enrolled in Medicine and Allied Health programs, 32% from students enrolled in Biology and Biotechnology programs, and 14% from students enrolled in Pharmaceutical programs. Students from Sports Science programs accounted for another 8% of the respondents.

**Fig. 2:**
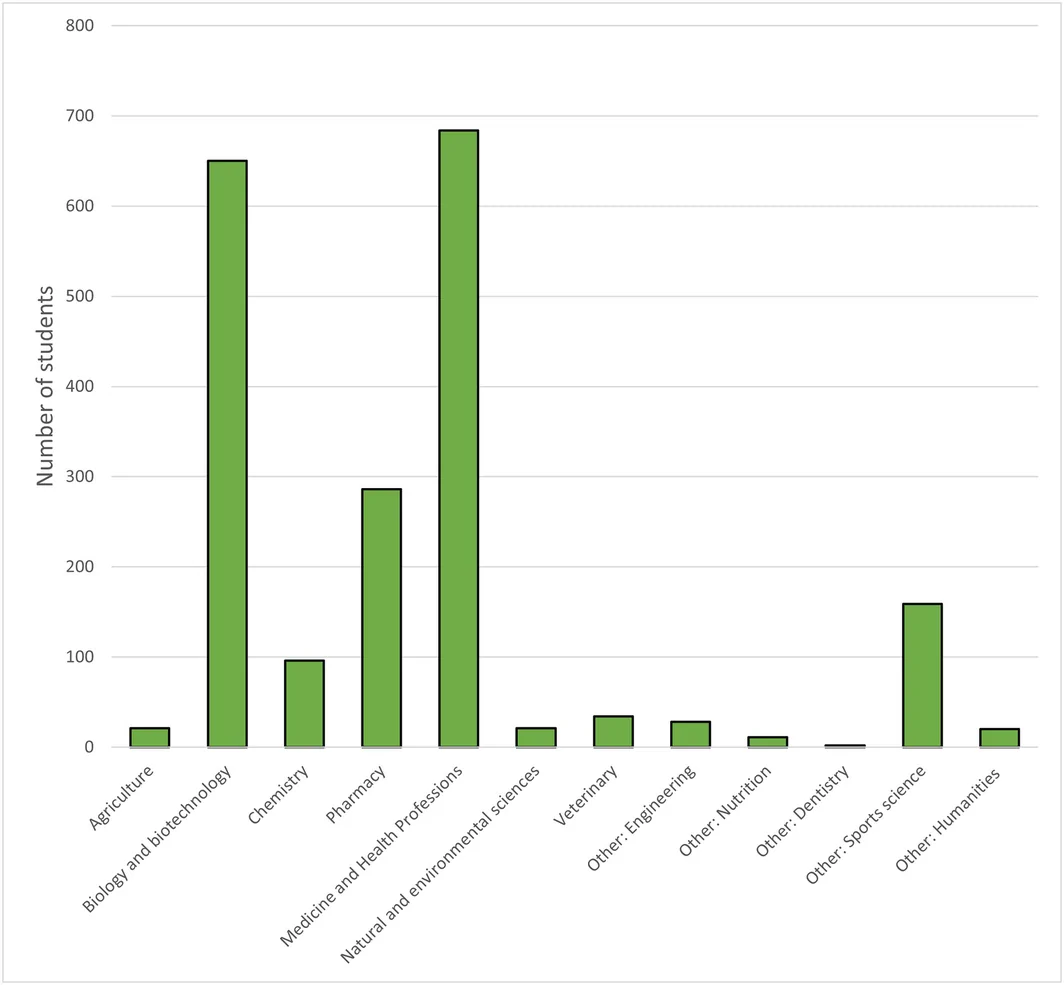
Distribution of the responders across different scientific areas. Bars report absolute numbers.

For the subsequent data analysis, we considered only respondents who had either already passed the examination (*n* = 888, i.e., Table 2, groups A1 + A2) or were actively preparing for the examination at the time of completing the questionnaire (*n* = 795, i.e., Table 2, group A3); this latter group also includes students who had previously sat the examination but had not passed it. Accordingly, our subsequent analysis focused on these 1683 students. Representing more than 80% of the total respondents (Fig. 3, yellow/orange slices of the pie chart), this sample was deemed adequate for subsequent statistical analyses.

**Fig. 3:**
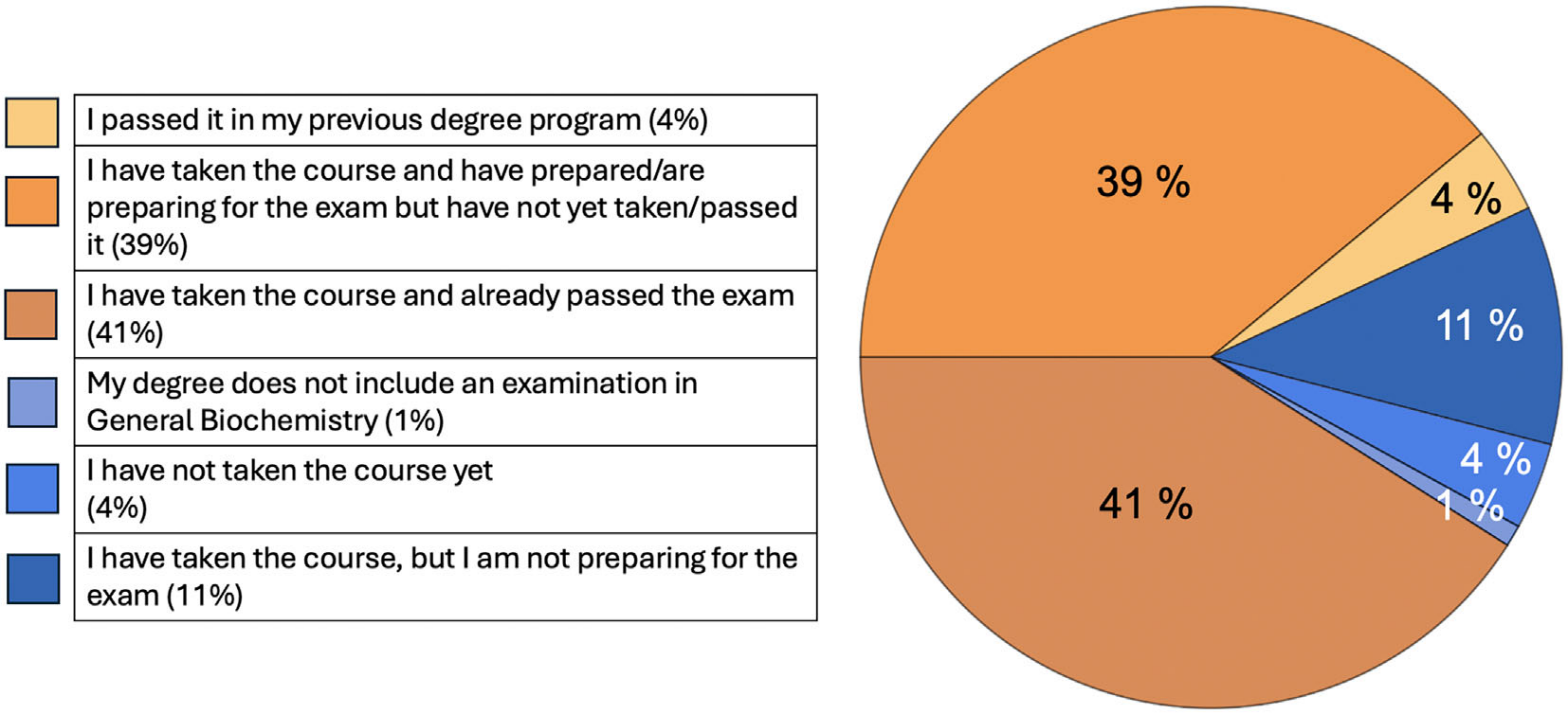
Percentage of responders who were included or excluded from the detailed analysis. The slices of the pie chart in orange shades with black numbers represent students who were considered for further questions, whereas the slices of the pie chart in blue shades with white numbers represent those students who were not considered for the rest of the questionnaire. Motivation for inclusion or exclusion is described in the text.

From the survey, it emerges that General Biochemistry courses in Italy are primarily mandatory, or at least strongly recommended, and are predominantly lecture‐based (72% of students state they have no laboratory activities planned for the course). Almost half (434/888; 49%) of the students who reported passing the examination declared a mark ≥ 28/30, which is considered ‘excellent’ in the Italian grading system. Since we noted that the marks reported by students in our questionnaire do not follow a normal distribution but are much more skewed toward higher values, we interpret this as indicating that students with nonexcellent marks were less inclined to complete the questionnaire. Nevertheless, 24% of respondents who passed the examinations declared low or very low marks (between 24/30 and 18/30), indicating that this group still contributed significantly to our survey.

Figure 4 shows the preferences for study materials in relation to the students' level of academic performance and/or examination preparation. We considered separately: students who passed the examination with a mark ≥ 28/30 (panel A), students who passed the examination with a mark < 28/30 (panel B), and students who are still actively preparing for the examination, including those who already failed it (panel C). In general, slides provided by the lecturer, personal notes, audio recordings of the lectures, and hard‐copy textbooks are the four preferred materials used by the students for their personal study, with varying degrees of preference across the different students' groups. Students who achieved marks ≥ 28/30 (Fig. 4A) indicated personal notes, followed by audio recordings and textbooks (hard copy+ebooks), as their preferred 1st‐choice material, while for the students who passed the examination with a mark < 28/30 (Fig. 4B), audio recordings, followed by personal notes and slides provided by the lecturer, were indicated as their preferred 1st choice. In general, the preferred 2nd‐choice material for all these groups of students was the slides provided by the teacher, which were overall (1st + 2nd + 3rd choice) the most used material. These patterns reflect differences in how students prioritize and organize their study resources.

**Fig. 4:**
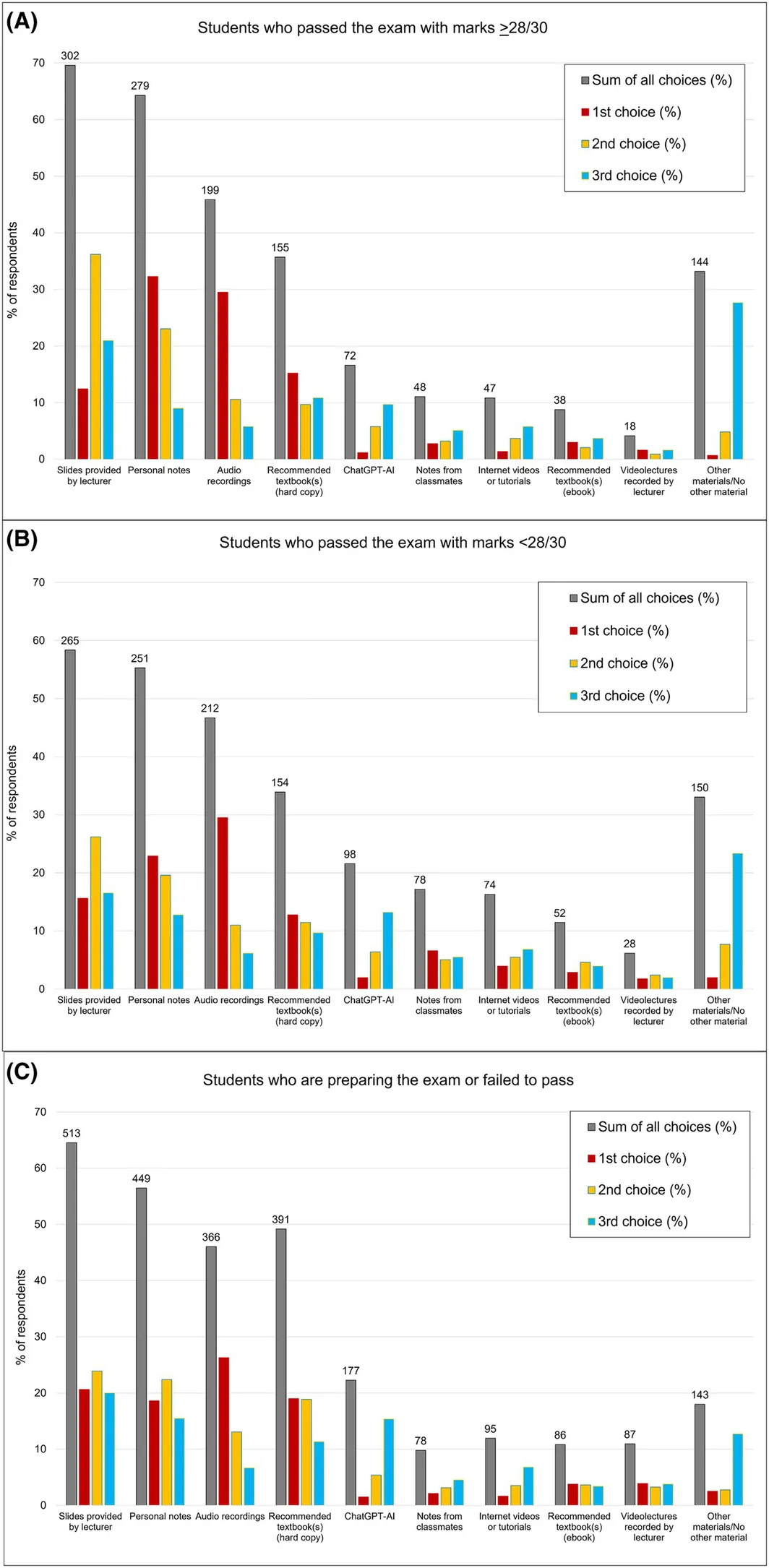
Percentage of preferred study materials used by students belonging to Group A (see Methods). Panel A: students who passed the examination with a mark ≥ 28/30; panel B: students who passed the examination with a mark < 28/30; panel C: students who are still preparing for the examination or failed to pass it. The gray bar indicates the sum of 1^st^ + 2^nd^ + 3^rd^ choices for each material; numbers on the gray bars indicate the total number of responses. The group “Other materials/No other material” aggregates the following items from the questionnaire: Other general biochemistry texts not recommended by the lecturer; E‐learning courses; Miscellaneous materials (notes, slides) downloaded from Internet sites; Other (specify); I did not use other materials.

For students who have not yet passed the examination (Fig. 4C), audio recordings, followed by slides provided by the lecturer and personal notes, are the preferred 1st‐choice materials. Also, for these students, slides are the 2nd preferred choice. Overall (1st + 2nd + 3rd choice), slides are the most used resource among this group of students as well, followed by personal notes and printed textbooks. However, when preferences for both printed and electronic versions are taken into account, textbooks become the second most used resource among students preparing for the examination, which indicates that a textbook is still seen as a valid reference for students' preparation.

Our questionnaire also highlighted students' use of artificial intelligence (AI)‐based tools. These tools are mainly indicated as 3rd‐choice material, but overall, their use ranks fifth, surpassing e‐book textbooks and other material downloaded from the internet in all the groups examined.

We examined whether the differences in study materials rankings among these student groups were statistically significant, revealing a consistent link between resource prioritization and students' preparation (Table 2, group A). The strongest effect in the entire study emerged among students who passed the examination with marks ≥ 28/30 (Table 2, group A1), where the association between rank position and material preference was particularly pronounced; notably, 32.5% of these students placed personal notes as their 1st choice (Fig. 4A). Students achieving marks < 28/30 showed an intermediate pattern (Table 2, group A2), with audio recordings leading as the 1st choice, slides as the 2nd choice, and other materials dominating as the 3rd choice. In contrast, students who have not yet passed the examination (Table 2, group A3) showed material preferences distributed more evenly across rank positions, suggesting that those students adopt a more exploratory and less consolidated study strategy. Importantly, the monotonic Cramér's *V* gradient across the three groups of students (Table 2) suggests an association between more structured prioritization of study resources and higher academic performance. From these data, actively taking notes during lessons seems to be more effective in terms of effective learning than passive listening and subsequently reworking the information provided by the teacher. However, other factors, such as motivation and metacognitive skills, may also influence both study strategies and academic performance, highlighting the need for further research to better clarify these relationships.

To determine whether preferences for study materials varied across scientific areas, we analyzed separately the responses of students enrolled in the three most represented degree programs: Biology, Medicine and Allied Health, and Pharmacy (Fig. 5A–C, respectively). Overall, *χ* ^2^ tests for Biology, Medicine, and Pharmacy students were statistically significant for all three rank positions, but the relatively low Cramér's *V* values demonstrate a weak‐to‐moderate effect size (Table 2, group B). This implies that the overarching rank order of the materials is more pronounced than the variations between student groups. While lecturer slides and students' own notes are generally considered important study material for all groups, medical students ranked audio recordings even higher, indicating it as the most important resource to prepare for the General Biochemistry examination. Moreover, students in Medicine and Allied Health programs tend to use AI‐based tools more frequently than other students, with 29% of them ranking such tools as one of their top three choices for studying, compared to 18% of Biology students and 12.5% of Pharmacy students (Fig. 5).

**Fig. 5:**
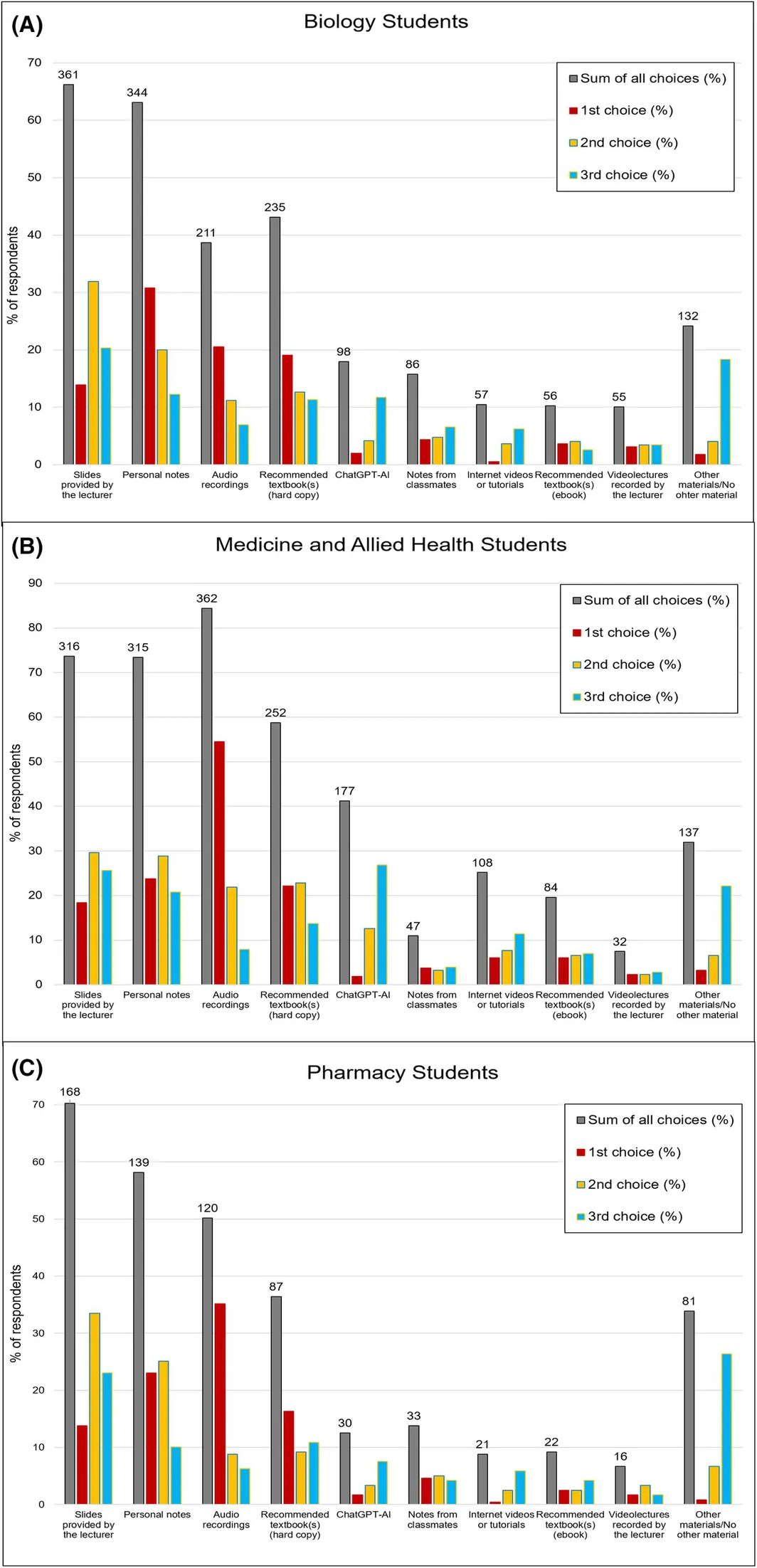
Percentage of preferred study materials used by students belonging to Group B (see Methods). Panel A: students enrolled in degrees in the area of Biology; panel B: students enrolled in degrees in the area of Medicine and Allied Health; panel C: students enrolled in degrees in the area of Pharmacy. The gray bar indicates the sum of 1^st^ + 2^nd^ + 3^rd^ choices for each material; numbers on the gray bars indicate the total number of responses. The group “Other materials/No other material” aggregates the following items from the questionnaire: Other general biochemistry texts not recommended by the lecturer; E‐learning courses; Miscellaneous materials (notes, slides) downloaded from Internet sites; Other (specify); I did not use other materials.

The pairwise statistical analysis performed on these results showed that the strongest and most consistent contrast emerges between Biology and Medicine students, which differ significantly across all three rank positions (Table 2, group B). This pattern remains consistent across 2nd‐ and 3rd‐choice resources, suggesting that the students enrolled in these two‐degree programs are characterized by fundamentally different study resource hierarchies. Comparisons between Biology and Pharmacy students showed a significant difference only for 1st‐choice materials (*V* = 0.166, corrected *P* = 0.030), mainly due to Pharmacy students' overall stronger preference for audio recordings (50.21% vs 38.72%, respectively, for Pharmacy vs Biology, see Fig. 5A,C). However, this last result should be interpreted cautiously because several cells in the underlying contingency table had expected counts below five (minimum = 1.22), which can potentially reduce the statistical power and reliability of the *χ* ^2^ test. Conversely, no significant differences were found between Biology and Pharmacy students for 2nd or 3rd choices, indicating broadly similar lower‐rank study preferences. In contrast, Medicine and Pharmacy students show no significant difference in first‐choice materials (both heavily favoring audio recordings), but differ substantially in 2nd and 3rd choices, with significant effects for both positions (*V* = 0.229 and 0.196, respectively, see Table 2). Notably, Medicine students placed ChatGPT‐AI more prominently in third position (18.9%), whereas Pharmacy students more often selected other materials (26.4%) and lecturer slides (23.0%) (Fig. 5B,C). Overall, among all degree programs, students in Medicine and Allied Health emerged as showing the most distinctive pattern in the study of Biochemistry in Italy, characterized by exceptionally high reliance on audio recordings as a primary resource and a uniquely prominent use of ChatGPT‐AI as a third‐choice resource.

For each study material selected in the questionnaire, students were also asked to explain their choice (data not shown). We analyzed the justifications given by students who had passed the examination with respect to their 1st‐choice material and found that the responses could be grouped into a few broad themes:

- audio recordings were selected because they enable direct access to the teacher's explanations, are perceived as a comprehensive overview, and provide guidance on the topics most frequently covered in examinations;
- lecturer slides, on the other hand, are used as a guide for structuring study sessions, are valued for their visual content (e.g., diagrams and charts), and complement students' own notes;
- personal notes are valued because they are customized to the student's level of understanding, provide comprehensive coverage, and promote active engagement during class;
- finally, textbooks are valued for clarity, comprehensiveness, and capacity to facilitate deeper exploration of the subject matter.

In order to verify how the course workload is managed by the students, our questionnaire asked them to provide a rough estimate of the daily time spent preparing for the final assessment and the number of weeks needed to complete the preparation. The responses outline mainly an intensive, short‐term approach to preparation, in which students focus on one or two subjects at a time, devoting many hours each day over a few weeks rather than distributing their efforts evenly across multiple courses throughout the semester.

The final section of the questionnaire used 5‐point Likert scales, ranging from 1 (‘low’) to 5 (‘high’), to assess students' perceived importance of biochemistry for their education, the role of class attendance in effective learning, and their enjoyment of the subject. As shown in Fig. 6, panels A and B respectively, 74% of the students attributed great importance (score ≥ 4) to the subject *per se*, and 72% to class attendance for achieving good learning outcomes in biochemistry. Notably, 60% of students also reported enjoying the study of biochemistry very much, regardless of their mastery of the material (Fig. 6C). When asked about the most difficult aspects of studying biochemistry, 55% of students reported finding biochemistry challenging primarily due to the large number of concepts that must be understood; 31% cited the complexity of the concepts as the main reason, while 14% of students specifically cited the need to learn all the material by heart as their primary challenge.

**Fig. 6:**
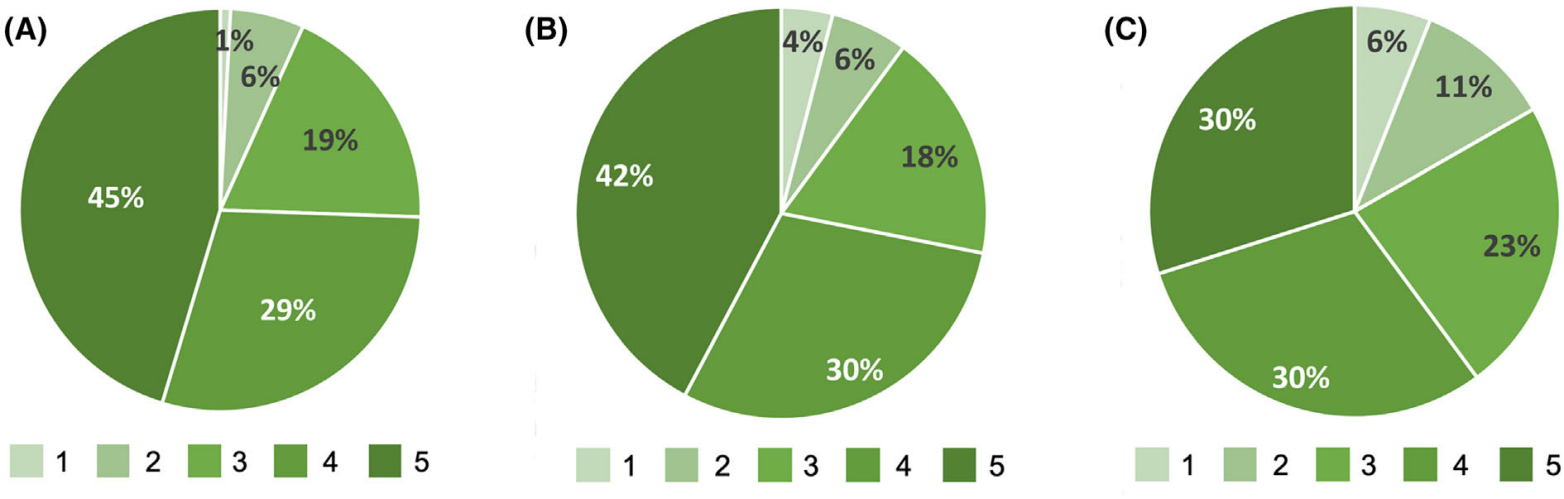
Students' perceived importance and satisfaction with biochemistry. Panel A: perceived importance of biochemistry for adequate preparation in the degree program. Panel B: perceived importance of attending the course for building a solid foundation in general biochemistry. Panel C: students' level of satisfaction with the subject. The scores from 1 (“low”) to 5 (“high”) rank the levels of importance or satisfaction in ascending order. Slices with higher scores (4 and 5) are highlighted with white fonts.

Finally, students were invited to suggest which elements, in their opinion, would make studying biochemistry more enjoyable. Again, their open‐ended responses (data not shown) can be summarized in a few key concepts:

- more laboratory activities;
- greater emphasis on the relevance of biochemical phenomena to real‐world contexts;
- reducing the course load by introducing mid‐term assessments;
- limiting excessive details and mnemonic‐heavy concepts in the final examination;
- adopting innovative teaching aids (though not specified in detail) to make explanations clearer.

It is worth noting that only a few anonymous criticisms were directed at the instructors. Moreover, an examination of students' statements revealed several comments suggesting that, with few exceptions, instructors are generally perceived as competent and empathetic toward students.

## Discussion

To the best of our knowledge, this is the first time in Italy that a questionnaire was administered to students to investigate their study habits in Biochemistry. The high number of responses from across Italy, together with the candor of the answers provided, suggests a genuine willingness among students to report their study habits. This may, in turn, reflect students' hope that their feedback could prompt biochemistry instructors in Italy to more effectively reconsider 21st‐century teaching methodologies and tools for a discipline that remains fascinating and engaging, despite its historical roots, and whose relevance in the era of new technologies is still fundamental.

The materials indicated by students as their main sources of information for learning are those traditionally expected to be used by students attending a university course, at least in Italy. However, our questionnaire highlights that traditional textbooks are not the primary source of knowledge acquisition, even among students who achieve high examination marks. Probably due to limited preparation time and the large amount of information to be acquired, students prefer to focus almost exclusively on the topics that are indicated as fundamental by teachers for passing the examination, and which are therefore presented and examined in greater depth in class. Students therefore mainly use the teachers' instructions, in the form of audio recordings of classroom lessons, slides provided by the teachers themselves, and personal notes, while using the textbook only to explore selected concepts in greater detail. The material that students prefer to use is therefore largely the result of re‐elaboration (either personal or by the teacher). Our findings highlight the importance of ensuring that instructor‐provided materials are accurate and clearly presented, given their central role in students' study strategies. One obvious consequence of this observation is that teachers must carefully verify the accuracy of the information presented in their slides and remain highly attentive when giving explanations to minimize the risk of conveying incorrect information as much as possible. Concerning personal notes, students find them useful both for staying focused in class and for carrying out an initial reworking of information, which is essential for active learning. This is clearly a positive aspect, as our statistical analysis has shown a higher correlation between the preference for this study material and a better performance of the students. However, the use of notes without other forms of control exposes them to the risk of inaccuracies. These considerations highlight the importance of guiding students in evaluating different sources of information. Teachers should also clarify the complementary roles of different sources, encouraging students to critically compare lecture materials with textbooks and other reference sources; in particular, in the event of discrepancies, students should be informed that the textbook is the most authoritative source to consult.

Regarding textbooks, students still prefer them in paper format, while e‐books, although often less expensive and richer in interactive content such as questionnaires, videos, and links to selected sites are not widely used in Italy. The latter seems paradoxical, considering that students, in their open‐ended responses, repeatedly suggested that teachers use additional teaching aids during lessons. Therefore, teachers should probably strive to prepare lessons that make the most of this supplementary material and be the first to use it in class, thereby encouraging students to use it as well. Publishers could help teachers in this regard, for example, by organizing seminars to illustrate these materials, in which teachers are guided on how to structure lessons in such a way as to make them more stimulating, engaging, and interactive. In addition, it may be useful to familiarize students with public‐domain scientific databases where they can readily access information that is useful for their studies. For example, when studying enzymes, it would be useful to introduce the BRENDA database (https://www.brenda‐enzymes.org/) [17] and to explain protein structures by showing the PDB database (https://www.wwpdb.org/) [18].

As was inevitable, AI‐based tools are significantly modifying teaching and learning practices and are beginning to play a relevant role in biochemistry education. Although it is impossible to prevent students from using these tools, teachers should nevertheless advise them not to treat AI tools as an ‘oracle’ that provides quick, ready‐to‐use information to be accepted uncritically. They should also explain that the information provided by AI tools may be inaccurate or that its sources cannot always be traced. Indeed, it is widely documented that, under some circumstances, the so‐called AI hallucinations occur. In these cases, generative AI produces information that seems plausible but is rather incorrect or fabricated [19]. Hallucinations frequently occur, especially when AI models are prompted to provide citations and to find appropriate sources. This phenomenon could be particularly dangerous for students who are less confident in identifying sources that lack scientific soundness. For example, teachers should encourage students to compare what the most common AI tools, such as ChatGPT and Gemini, report on a given topic with the original sources and/or textbooks, and to verify the accuracy of the information received. This highlights the need for explicit guidance on the appropriate use of AI tools, in line with emerging literature on AI literacy in higher education [20]. Moreover, teachers could potentially train chatbots to provide better guidance to students and upload reliable knowledge bases for the systems to use in their responses.

Further analysis of the responses to our survey suggests that students wish to experience biochemistry as a discipline closely related to their interests and everyday life. It is therefore essential that teachers select practical examples that meet this expectation and customize them to the context of the degree program in which biochemistry is taught. For example, students entering the medical profession must understand how biochemistry provides the knowledge, skills, and abilities to comprehend most diseases. This reinforces the idea that diseases affecting organs or tissues are primarily linked to molecular alterations, which can only be fully understood by studying the biochemical processes that are disrupted as a result. For students enrolled in Pharmacy degree programs, it is essential to show that the development of drugs is rooted in their interactions with biological macromolecules, whose characteristics and activities are studied in biochemistry. Moreover, biology students should be helped to perceive biochemistry as the discipline underlying the functioning of the most diverse living organisms, emphasizing the commonality of biochemical phenomena in the most diverse forms of life, from bacteria to higher organisms. And, of course, equally important is giving students the opportunity to experience biochemical phenomena firsthand through educational laboratory activities, which are more likely than lectures alone to stimulate curiosity and foster genuine interest in the discipline. Unfortunately, according to the questionnaire results, General Biochemistry courses in Italy still rely predominantly on lecture‐based teaching. This could be due to the inadequacy of the educational laboratories available to teachers, which are often small and unable to accommodate the large number of students attending the first years of the course study (typically those in which Biochemistry is taught), and to the shortage of teachers and support staff, which makes it very difficult for teachers (overburdened with courses to deliver) to carry out laboratory activities. To overcome these difficulties, it may nevertheless be useful to supplement lectures with multimedia materials showing laboratory activities relevant to the phenomena being explained, or to carry out simple demonstrations in the classroom that are effective without requiring the use of special equipment. In addition, virtual laboratory or simulation environments can be integrated not as substitutes for real laboratory practice, which remains essential for developing the hands‐on skills that underpin advanced competencies, but as complementary tools that broaden, extend, reinforce and personalize the laboratory experience at the student's own pace.

Another suggestion concerns the sheer volume of information that anyone studying Biochemistry inevitably must deal with. In a world where basic knowledge is readily accessible through digital devices, students increasingly perceive the memorization of names and reactions of metabolic pathways as unnecessary and outdated. What students need instead is to understand the logic, efficiency, and biological significance underlying these processes, so that biochemical knowledge becomes meaningful rather than merely mnemonic.

Finally, we would like to point out that, despite problems and limitations, biochemistry is still perceived by most students as a fascinating subject and a pillar for the preparation of future professionals in various fields of life sciences. Indeed, the study of biochemistry reveals the molecular mechanisms that sustain life, from metabolism to genetic regulation. It helps us understand diseases at their molecular bases and develop innovative therapies and technologies. Moreover, biochemistry is a continuously evolving discipline that connects chemistry, biology, and medicine in addressing global challenges such as health and sustainability. Instructors should be able to convey to students that understanding these mechanisms empowers them to contribute actively to medical and technological innovation. A solid understanding of biochemistry fosters the critical thinking skills required to address complex biological problems.

## Conclusions

Our present work examines how university students in Italy study biochemistry and which educational materials they use. Our survey investigated not only the types of learning resources most commonly adopted by students but also the reasons behind the students' choices and their perception of biochemistry within their academic paths. Our analyses together paint an interesting and coherent picture. Study material choice hierarchies are not random: structured preferences vary as a function of rank position, suggesting that the ability to define clear study priorities is a behavioral correlate of academic success in biochemistry. Disciplinary differences, while genuine and statistically significant, exhibit only modest effect sizes, particularly in comparison with the much stronger effect of rank position on students' preferences.

By analyzing students' study habits and learning behaviors, our project provides valuable insights to support critical reflection on existing teaching approaches. In the future, it would be interesting to expand this survey to other countries in Europe to compare possible differences in students' study habits and gain further insights into the development of innovative biochemistry teaching materials that could be shared at a supranational level. In this perspective, our findings could contribute to the development of more engaging and accessible teaching materials and help align instructional practices with students' needs and expectations. In addition, digital learning environments may offer opportunities to support student engagement and more flexible learning experiences. Overall, this study aims to support the development of teaching strategies for our students that are more effective, engaging, and inclusive, and that support a deeper and more lasting understanding of biochemistry.

## Conflict of interest

The authors declare no conflict of interest.

## Author contributions

AM conceived the project, designed the draft of the survey, and drafted the manuscript; FMa performed the statistical analyses; all authors contributed in survey finalization and dissemination, data interpretation, and manuscript preparation.

## Data Availability

The answers to the survey, available in Italian (1885 answers) and in English (127 answers), can be requested from the Authors and viewed in aggregate form to preserve the anonymity of the responses.
